# Supplementary material for: Application of the double bounded dichotomous choice model to the estimation of parent’s willingness to pay for the hand foot mouth disease vaccination: A survey in Selangor, Malaysia
Source: PLoS One. 2023 Jun 12;18(6):e0286924. doi: 10.1371/journal.pone.0286924 (PMC10259800; doi:10.1371/journal.pone.0286924)
Supplement: S1 File — (PDF) [file pone.0286924.s001.pdf]

Willingness to pay for Hand Foot Mouth Diseases (HFMD) in Selangor, Malaysia

District: ___________________

Date: ________________

INSTRUCTIONS: Answer all questions and tick (√) in each answer.

Part A: Socioeconomics

1. Age : ________________ years old

2. Gender:

   ( ) Male ( ) Female

3. Race:

   ( ) Malay ( ) Chinese ( ) Indian ( ) Others: _________

4. Marital Status:

   ( ) Single ( ) Married ( ) Divorced ( ) Widow / widower

5. Education:

   ( ) High School or below ( ) Certificate or Diploma

   ( ) Bachelor's Degree ( ) Postgraduate Education

6. Occupation:

   ( ) Public sector ( ) Private sector ( ) Self-employed ( ) Student

   ( ) Others: ______________

7. Household Monthly Income: ____________________ RM per month

Part B: Willingness to pay

The hypothetical HFMD vaccine was described to respondents as being safe (no danger of becoming infected from the vaccine), having no side-effects, comprising two doses (second dose given after 1 month), able to prevent HFMD infection among those who are not yet infected (but having no benefit for someone already infected), effective for 5 years.

8. Based on the scenario above, are you willing to vaccinate your kids with the vaccine?

( ) Yes (go to 9)

( ) No (Stop answering)

9. Based on your current income, you are willing to pay for HFMD vaccine if the cost of vaccines is **<u>RM100/RM200/RM300/RM400</u>** for TWO injections?

( ) Yes (go to 11)

( ) No (go to 10)

10. What if the cost is reduced to **<u>RM50/RM150/RM250/RM350</u>** for TWO injections, are you willing to pay?

( ) Yes

( ) No

11. What if cost increased to **<u>RM150/RM250/RM350/RM450</u>** for TWO injections, are you willing to pay?

( ) Yes

( ) No
