# Supplementary material for: Application of the double bounded dichotomous choice model to the estimation of parent’s willingness to pay for the hand foot mouth disease vaccination: A survey in Selangor, Malaysia
Source: PLoS One. 2023 Jun 12;18(6):e0286924. doi: 10.1371/journal.pone.0286924 (PMC10259800; doi:10.1371/journal.pone.0286924)
Supplement: Attachment — Submitted filename: PONE-D-22-33005_reviewer.pdf [file pone.0286924.s002.pdf]

PLOS ONE

Application of the Double Bounded Dichotomous Choice Model to the Estimation of Parent's Willingness to pay for the Hand Foot Mouth Disease Vaccination: A survey in Selangor, Malaysia

--Manuscript Draft--

**Manuscript Number:**	PONE-D-22-33005
**Article Type:**	Research Article
**Full Title:**	Application of the Double Bounded Dichotomous Choice Model to the Estimation of Parent's Willingness to pay for the Hand Foot Mouth Disease Vaccination: A survey in Selangor, Malaysia
**Short Title:**	Parent's Willingness to pay for the Hand Foot Mouth Disease Vaccination
**Corresponding Author:**	Yogambigai Rajamoorthy, Ph.D<br>Universiti Tunku Abdul Rahman<br>Kajang, Selangor MALAYSIA
**Keywords:**	Willingness to pay, Hand Foot Mouth Disease, vaccine , Malaysia
**Abstract:**	Hand foot and mouth disease (HFMD) is a notifiable viral disease in Malaysia, and is transmitted primarily among young children. Although vaccines for enteroviruses 71 (EV-71) were approved in China against HFMD, the availability and the acceptance of the vaccine in the Malaysia are unknown. This study investigated and ascertained the determinants of willingness-to-pay (WTP) for HFMD vaccination in Selangor Malaysia. This study adopted a cross-sectional, contingent valuation method involving 390 parents of young children aged six and below. The double bounded dichotomous choice (DBDC) approach was employed to assess the WTP for HFMD vaccine among respondents. A bivariate probit model was used to assess the key determinants of WTP for HFMD vaccine, while the mean WTP was measured using the Krinsky and Robb procedure. We found that 279 (71.5%) of parents were willing to pay for the HFMD vaccination. The estimated single bounded mean WTP was MYR460.23 (equivalent to US$ 102.17) for two doses of HFMD vaccination. The double bounded analysis revealed that the vaccine's price, poor education background and lower income were the key factors that significantly affected the WTP, with the estimated mean WTP being MYR394.00 (US$ 87.47). In conclusion, most Malaysian parents are willing to pay for the HFMD vaccination. The estimated WTP identifies the optimal price point for HFMD vaccination in Malaysia. Furthermore, the government should focus on an awareness programme for the HFMD vaccination among parents who earn lower incomes and illiteracy.
**Order of Authors:**	Yogambigai Rajamoorthy, Ph.D
	Niazlin Mohd Taib
	Harapan Harapan
	Abram Luther Wagner
	Subramanaim Munusamy
**Additional Information:**	
**Question**	**Response**
**Financial Disclosure**<br><br>Enter a financial disclosure statement that describes the sources of funding for the work included in this submission. Review the submission guidelines for detailed requirements. View published research articles from *PLOS ONE* for specific	The funders had no role in study design, data collection and analysis, decision to publish, or preparation of the manuscript

Powered by Editorial Manager® and ProduXion Manager® from Aries Systems Corporation

examples.<br><br>This statement is required for submission and **will appear in the published article** if the submission is accepted. Please make sure it is accurate.<br><br>**Unfunded studies**<br>Enter: *The author(s) received no specific funding for this work.*<br><br>**Funded studies**<br>Enter a statement with the following details:<br>• Initials of the authors who received each award<br>• Grant numbers awarded to each author<br>• The full name of each funder<br>• URL of each funder website<br>• Did the sponsors or funders play any role in the study design, data collection and analysis, decision to publish, or preparation of the manuscript?<br>• **NO** - Include this sentence at the end of your statement: *The funders had no role in study design, data collection and analysis, decision to publish, or preparation of the manuscript.*<br>• **YES** - Specify the role(s) played.<br><br>* typeset	
**Competing Interests**<br><br>Use the instructions below to enter a competing interest statement for this submission. On behalf of all authors, disclose any competing interests that could be perceived to bias this work—acknowledging all financial support and any other relevant financial or non-financial competing interests.<br><br>This statement is required for submission and **will appear in the published article** if the submission is accepted. Please make sure it is accurate and that any funding sources listed in your Funding Information later in the submission form are also declared in your Financial Disclosure statement.	The authors have declared that no competing interests exist

Powered by Editorial Manager® and ProduXion Manager® from Aries Systems Corporation

View published research articles from *PLOS ONE* for specific examples.<br><br>**NO authors have competing interests**<br><br>Enter: *The authors have declared that no competing interests exist.*<br><br>**Authors with competing interests**<br><br>Enter competing interest details beginning with this statement:<br><br>*I have read the journal's policy and the authors of this manuscript have the following competing interests: [insert competing interests here]*<br><br>* typeset	
**Ethics Statement**<br><br>Enter an ethics statement for this submission. This statement is required if the study involved:<br><br>• Human participants<br>• Human specimens or tissue<br>• Vertebrate animals or cephalopods<br>• Vertebrate embryos or tissues<br>• Field research<br><br>Write "N/A" if the submission does not require an ethics statement.<br><br>General guidance is provided below. Consult the submission guidelines for detailed instructions. **Make sure that all information entered here is included in the Methods section of the manuscript.**	The Scientific and Ethical Review Committee of Universiti Tunku Abdul Rahman approved this study protocol (approval U/SERC/17/2020).

Powered by Editorial Manager® and ProduXion Manager® from Aries Systems Corporation

**Format for specific study types**<br><br>**Human Subject Research (involving human participants and/or tissue)**<br>• Give the name of the institutional review board or ethics committee that approved the study<br>• Include the approval number and/or a statement indicating approval of this research<br>• Indicate the form of consent obtained (written/oral) or the reason that consent was not obtained (e.g. the data were analyzed anonymously)<br><br>**Animal Research (involving vertebrate animals, embryos or tissues)**<br>• Provide the name of the Institutional Animal Care and Use Committee (IACUC) or other relevant ethics board that reviewed the study protocol, and indicate whether they approved this research or granted a formal waiver of ethical approval<br>• Include an approval number if one was obtained<br>• If the study involved *non-human primates*, add *additional details* about animal welfare and steps taken to ameliorate suffering<br>• If anesthesia, euthanasia, or any kind of animal sacrifice is part of the study, include briefly which substances and/or methods were applied<br><br>**Field Research**<br><br>Include the following details if this study involves the collection of plant, animal, or other materials from a natural setting:<br>• Field permit number<br>• Name of the institution or relevant body that granted permission	
**Data Availability**<br><br>Authors are required to make all data underlying the findings described fully available, without restriction, and from the time of publication. PLOS allows rare exceptions to address legal and ethical concerns. See the PLOS Data Policy and FAQ for detailed information.	Yes - all data are fully available without restriction

Powered by Editorial Manager® and ProduXion Manager® from Aries Systems Corporation

A Data Availability Statement describing where the data can be found is required at submission. Your answers to this question constitute the Data Availability Statement and **will be published in the article**, if accepted.<br><br>**Important:** Stating 'data available on request from the author' is not sufficient. If your data are only available upon request, select 'No' for the first question and explain your exceptional situation in the text box.<br><br>Do the authors confirm that all data underlying the findings described in their manuscript are fully available without restriction?	
**Describe where the data may be found in full sentences. If you are copying our sample text, replace any instances of XXX with the appropriate details.**<br><br>• If the data are **held or will be held in a public repository**, include URLs, accession numbers or DOIs. If this information will only be available after acceptance, indicate this by ticking the box below. For example: *All XXX files are available from the XXX database (accession number(s) XXX, XXX.)*.<br>• If the data are all contained **within the manuscript and/or Supporting Information files**, enter the following: *All relevant data are within the manuscript and its Supporting Information files.*<br>• If neither of these applies but you are able to provide **details of access elsewhere**, with or without limitations, please do so. For example:<br><br>*Data cannot be shared publicly because of [XXX]. Data are available from the XXX Institutional Data Access / Ethics Committee (contact via XXX) for researchers who meet the criteria for access to confidential data.*<br><br>*The data underlying the results presented in the study are available from (include the name of the third party*	The data underlying the results presented in the study are available from https://figshare.com/s/3e6dd659b5efbc7a5e65

and contact information or URL).<br>• This text is appropriate if the data are owned by a third party and authors do not have permission to share the data.<br><br>* typeset	
Additional data availability information:	Tick here if the URLs/accession numbers/DOIs will be available only after acceptance of the manuscript for publication so that we can ensure their inclusion before publication.; Tick here if your circumstances are not covered by the questions above and you need the journal's help to make your data available.

Powered by Editorial Manager® and ProduXion Manager® from Aries Systems Corporation

Manuscript Click here to access/download;Manuscript;manuscript.docx

Application of the Double Bounded Dichotomous Choice Model to the Estimation of Parent's Willingness to pay for the Hand Foot Mouth Disease Vaccination: A survey in Selangor, Malaysia

Yogambigai Rajamoorthy[1,*], Niazlin Mohd Taib[2], Harapan Harapan[3,4,5], Abram Luther Wagner[6], Subramaniam Munusamy[7]

[1] Department of Economics, Faculty of Accountancy and Management, Universiti Tunku Abdul Rahman, Sungai Long Campus, Jalan Sungai Long, Cheras, 43000 Kajang, Selangor, Malaysia

[2] Department of Medical Microbiology and Parasitology, Faculty of Medicine and Health Science, Universiti Putra Malaysia, UPM Serdang, Selangor, Malaysia

[3] Department of Microbiology, School of Medicine, Universitas Syiah Kuala, Banda Aceh 23111, Indonesia

[4] Medical Research Unit, School of Medicine, Universitas Syiah Kuala, Darussalam, Banda Aceh 23111, Indonesia

[5] Tropical Disease Centre, School of Medicine, Universitas Syiah Kuala, Banda Aceh 23111, Indonesia

[6] Department of Epidemiology, University of Michigan, Ann Arbor, MI, USA

[7] Faculty of School of Management and Business, Manipal International University, 71800 Putra Nilai, Negeri Sembilan, Malaysia

*Corresponding author: yogambigai@utar.edu.my (YR)

Abstract

Hand foot and mouth disease (HFMD) is a notifiable viral disease in Malaysia, and is transmitted primarily among young children. Although vaccines for enteroviruses 71 (EV-71) were approved in China against HFMD, the availability and the acceptance of the vaccine in the Malaysia are unknown. This study investigated and ascertained the determinants of willingness-to-pay (WTP) for HFMD vaccination in Selangor Malaysia. This study adopted a cross-sectional, contingent valuation method involving 390 parents of young children aged six and below. The double bounded dichotomous choice (DBDC) approach was employed to assess the WTP for HFMD vaccine among respondents. A bivariate probit model was used to assess the key determinants of WTP for HFMD vaccine, while the mean WTP was measured using the Krinsky and Robb procedure. We found that 279 (71.5%) of parents were willing to pay for the HFMD vaccination. The estimated single bounded mean WTP was MYR460.23 (equivalent to US$ 102.17) for two doses of HFMD vaccination. The double bounded analysis revealed that the vaccine's price, poor education background and lower income were the key factors that significantly affected the WTP, with the estimated mean WTP being MYR394.00 (US$ 87.47). In conclusion, most Malaysian parents are willing to pay for the HFMD vaccination. The estimated WTP identifies the optimal price point for HFMD vaccination in Malaysia. Furthermore, the government should focus on an awareness programme for the HFMD vaccination among parents who earn lower incomes and illiteracy.

Introduction

Hand foot and mouth disease (HFMD) is transmissible from person to person by direct contact with nose and throat discharges, saliva, fluid from blisters, or from the stool of infected persons. Young children, especially in crowded areas such as in childcare centres, have a high-risk encountering and spreading the disease [1]. HFMD outbreaks result in closure of childcare centres, which entails secondary costs of parental absenteeism from work [2]. Cases of disease could have other, often unrecognized, impacts on the socioeconomic status of a population [3]. It is also possible for more virulent strains of the virus – in terms of neurological complications and mortality – to emerge in the future [4].

Enterovirus 71 (EV-71) is the primary causative agent for HFMD epidemics. The infection is associated with worldwide public health issues with a probability of pandemic emergence [4]. The first epidemic of HFMD in Malaysia was in 1997 followed by a subsequent outbreak in late 2000, in 2003, 2005, 2006, 2010 and 2018. Each caused multiple deaths amongst young children [4]. Since 2015, three inactivated EV-71 vaccines have been approved in China [3]. However, the vaccines have not yet been approved in Malaysia. Parental willingness to accept the EV-71 vaccines will be affected by its efficacy, side effects, and cost. There are only two studies published in China on the willingness of parents to vaccinate [3,5]. However, the willingness to pay (WTP) for HFMD vaccination has yet to be estimated, and could also differ between countries based on their epidemiological background and population experiences with disease. Therefore, this study's main objective was to estimate WTP of parents for HFMD and identify its determinants by employing the contingent valuation method in Selangor state.

Materials and methods

Ethics approval

The Scientific and Ethical Review Committee of Universiti Tunku Abdul Rahman approved the study protocol (approval U/SERC/17/2020). A brief explanation of the study was given to all participants and written informed concern was obtained from all participants.

Study design and setting

The sample size and respondents' selection criteria were a part of a Malaysian HFMD Project that have been described elsewhere [6,7]. A cross-sectional survey was conducted in Selangor state, which encircles the capital Kuala Lumpur. To represent the population, several kindergartens from each district were randomly selected. The Krejcie and Morgan sampling method was adapted to determine the study sample size [8]. As a minimal sample size, 384 participants were required on the following assumptions: (a) 5% margin of error; and (b) 95% confidence level. The participants were selected using a three-stage cluster sampling method. Using the nine administrative districts in Selangor as a sampling frame, the number of samples from each district was calculated based on its population size proportion (i.e. high numbers in some districts and low in some districts). In the second stage, a convenience sample of kindergartens was selected based on kindergarten lists in each district. In the last stage, a convenience sample of participants was recruited from each kindergarten using a quota to meet each district's calculated sample size. Before starting the study, we doubled the sample size to 770 to avoid insufficient sample size due to incomplete data.

The English version questionnaire was developed based on the existing literature and translated to Malay language. The questionnaire used in this study is given in S1 file. A panel consisting of a medical microbiologist and a public health doctor were appointed to validate the

questionnaire content in both version. The finalised questionnaire was tested in pilot study of 100 respondents selected via a convenience sample.

This study adapted a double-bounded dichotomous choice (DBDC) format by using a questionnaire for the statistical efficiency suggested by previous studies [9-11]. The DBDC format of this study is summarized in Fig 1.

Figure 1

Respondents were provided a randomly chosen bid amount with a scenario where the hypothetical HFMD vaccine was considered safe (no danger of becoming infected from the vaccine), having no side-effects, comprising two doses (second dose given after one month), able to prevent HFMD infection among those who are not yet infected (but having no benefit for someone already infected) and being effective for five years. If the respondents answered "yes" to the bid amount, the amount was increased by MYR50 (equal to US$ 11.10 using a November 2022 exchange rate). However, if the respondent's response was "no" to the initial amount, the subsequent bid was decreased by MYR50 (US$11.10).

Statistical analysis

A bivariate probit model was used to assess the key determinants of HFMD vaccine and the mean WTP was measured using the Krinsky and Robb procedure [12].

Mathematically, the double bounded format represented in the following equation:

$$z_1^* = \delta_1\beta_1 + \varepsilon_1 \quad (1)$$

$$z_2^* = \delta_2\beta_2 + \varepsilon_2 \quad (2)$$

Both error terms (ε_1 , ε_2) in equation (1) and (2) are jointly and assumed to be normally distributed. The δ_1 is a vector of the predictor variables common to both outcomes with estimators β. The δ_1 and = δ_2 which might affect the parents WTP for HFMD vaccination.

Observing the two binary outcomes is expected;

$$z_1 = 1 \text{ if } z_1^* > 0 \text{ and } z_1 = 0 \text{ if } z_1^* \leq 0; \quad (3)$$

$$z_2 = 1 \text{ if } z_2^* > 0 \text{ and } z_2 = 0 \text{ if } z_2^* \leq 0; \quad (4)$$

According to Alberini [11], estimates of the bivariate probit model are preferred to that of the interval data logit. The correlation coefficient between the two consecutive bid error terms is close to zero. Therefore, after checking the correlation coefficients for the first and subsequent responses, the study used a bivariate probit model. To estimate a bivariate probit model, the mean of the initial question error term and the second follow up question error term are assumed independent and covariances are assumed zero [13]. Let P^1 be the first bid price and P^2 the second bid price. The WTP bounded in: $P^1 \leq WTP < P^2$ for the yes-no responses; $P^1 > WTP \geq P^2$ for the no-yes response; $WTP \geq P^2$ for a yes-yes response; and $WTP < P^2$ for no-no response. This study assumed the WTP of the respondents between the lowest (WTP_i^L) and the highest value (WTP_i^H). In this study, the mean WTP was calculated using the Krinsky-Robb method as follows:

$$\text{Mean} = \frac{-\bar{X}\beta'}{\beta_0} \quad (5)$$

Where, $\bar{X}$ is the row vector of the sample, including 1 for the constant term. The β' is the column vector of estimated coefficients. The β_0 is the coefficient on the bid variable. All the statistical analysis performed using STATA 13 and the significance was assessed using alpha 0.05.

Results

Participant characteristics

We received 690 participant responses during the study period between 13 January 2020 and 16 March 2020 and 248 data were excluded from the final analysis due to missing information. A total of 442 (64.05%) participants, well-distributed from regions of the district resulted. However, 52 respondents were eliminated due not being willing to pay for HFMD and the remaining 390 were analysed. Table 1 shows the parents' profile, where 272 (70.26%) were the mother, and 116 (29.74%) were the father. Most of the parents were aged 31 to 40 (72.05%) with a small proportion aged 51 and 60 (1.79%). Most respondents were Malay (64.87%) followed by Chinese (23.85%) and Indian (10.51%). Around 41% of the parents had a bachelor's degree qualification, and 21.28% had only a high school education or below. Of note, 32.56% of the parent's household income was RM7000 and above, and only 3.33% were RM1000 and below.

Table 1: Description of socio-economics variables

Characteristics	Frequency (N)	%
**Gender**		
Male	116	29.74
Female	274	70.26
**Age**		
20-30	29	7.44
31-40	281	72.05
41-50	73	18.72
51-60	7	1.79
**Race**		
Malay	253	64.87
Chinese	93	23.85
Indian	41	10.51
Others	3	0.77
**Education**		
High School or below	83	21.28
Certificate or Diploma	121	31.03
Bachelor’s Degree	158	40.51
Postgraduate Education	28	7.18
**Household income**		
<RM1000	13	3.33
RM1001-RM3000	106	27.69
RM3001-RM5000	101	25.90
RM5001-RM7000	41	10.51

RM7001 & above	127	32.56

The distribution of the double-bounded dichotomous choice responses shows that the higher the initial bids, the lower the percentage of YES-YES percentage and the percentage of NO-NO also increases (Table 2). For some parents, if they accept the initial bids, the higher bids will be given and vice versa. Interestingly, 10.8% of the parents rejected the price increase and 17.8% accepted reduced bids.

Table 2: The distribution of the dichotomous responses

**Initial bids**	**Yes-Yes**	**Yes-No**	**No-Yes**	**No-No**	**Observation**
RM100 (USD $22.20)	40 (66.6%)	3	12	6 (9.8%)	61

RM150<br>(USD $33.30)	32 (52.5%)	6	16	7 (11.5%)	61
RM200<br>(USD $44.40)	32 (48.5%)	11	10	13 (19.7%)	66
RM250<br>(USD $55.50)	28 (50%)	3	8	17 (30.4%)	56
RM300<br>(USD $66.60)	17 (35.4%)	3	12	16 (33.3%)	48
RM350<br>(USD $77.70)	32 (49.2%)	8	8	17 (26.1%)	65
RM400<br>(USD $88.80)	13 (39.4%)	8	3	9 (27.3%)	33
Total	194 (49.7%)	42 (10.8%)	69 (17.8%)	85 (21.8%)	390

This study used a bivariate probit model to estimate the WTP based on the error correction between the first and second responses for the bid's questions. The correlation between first responses for the WTP question and the second WTP question had a result of 0.39 and was statistically significant at 1%. This indicates that the separate probit model will not be efficient to estimate the WTP value [13]. Furthermore, the Rho correlation coefficient being statistically significantly indicated that the bivariate probit models are appreciated [15]. A Variance Inflation Factor (VIF) was computed to check the existence of serious multicollinearity problems among continuous and discrete explanatory variables. The result indicated no multicollinearity problem exists among the explanatory variables with a mean VIF was 3.27.

The bivariate probit model was used to identify explanatory variables that influence parents' WTP. As suggested by Mitchell and Carson [16] , a robust estimator was used to control the potential bias of non-normality and outliers in contingent valuation studies. This study used bivariate probit robust estimation to reduce the effects of heteroscedasticity.

The relationship between the responses for single and double bounded choices with the independent variables was checked using a chi-square test. The Chi-square test was used to test the variable having a relationship with the response's variables. The result of chi-square is shown in Table 3. Only income and education associate with single and double bounded responses.

Table 3: Chi-square test of association between the responses (single and double bounded) and each explanatory variable

	Single bounded			Double bounded		
Explanatory variables	Chi-square	df	P value	Chi-square	df	P value
Gender	24.425	28	0.659	0.581	1	0.446
Age	0.524	1	0.469	34.224	28	0.194
Education level	26.477	3	0.000***	7.127	3	0.068*
Income	90.220	64	0.017**	79.381	64	0.093*
Ethnicity	2.918	3	0.404	3.381	3	0.337

***Statistically significant at 1%, **statistically significant at 5% and *statistically significant at 10%

The chi-square test of association, income and education variables is included in the univariate probit model (Table 4). The result shows that high school or below, certificate or diploma, bachelor’s degree, income ranged MYR1001 to 3000 and MYR7000 and above are significantly determine single bounded WTP. However, for double bounded, the determinants are found for parents with poor education background, high and low income.

Table 4: Probit Univariate Analysis

Explanatory variables	Single bounded			Double bounded		
	Parameter estimate	Std error	P value	Parameter estimate	Std error	P value
**Education**						
High School or below	-0.716	0.158	0.000***	-0.408	0.157	0.010***
Certificate or Diploma	0.311	0.142	0.028**	0.183	0.144	0.205
Bachelor’s Degree	0.348	0.133	0.009***	0.132	0.135	0.327
Postgraduate Education	-0.385	0.246	0.119	0.013	0.256	0.961
**Household income**						
<RM1000	-0.584	0.359	0.104	-0.987	0.370	0.008***
RM1001-RM3000	-0.412	0.143	0.004***	-0.201	0.146	0.168
RM3001-RM5000	-0.073	0.146	0.617	-0.114	0.149	0.445
RM5001-RM7000	0.306	0.218	0.160	0.359	0.229	0.118
RM7001 & above	0.411	0.144	0.004***	0.313	0.144	0.030**

*statistically significant at 10%
**statistically significant at 5%
***Statistically significant at 1%

Table 5 shows that education and income have an impact on the WTP**.** The coefficient value of the single bound, which is 0.001, shows that with a 1% increase in bid the probability of parents WTP reduces by 0.1%. However, the double bounded function indicates that with a 1% increase in the second bid, the probability of parents WTP reduces by 0.3%. This implies that the higher the price (bid) of the vaccine, the lower the WTP by the parents for the HFMD vaccine.

Single bounded responses show that educated parents have greater WTP for HFMD vaccination. A 1% increase in parents who have a certificate or diploma and bachelor's degree increases their WTP by 72.5% and 64.8%, respectively. However, double bounded responses shows that a 1% increase in parents who lack education reduces their WTP by 43.5%. This implies that educated parents have more WTP for HFMD vaccine compared to unenlightened parents.

In single bounded responses, a 1% increase in parents with high income will increase their WTP by 35.6%. However, in double bounded responses, a 1% increase in low-income parents will reduce their WTP by 71.6%. This suggests that higher parents' income significantly improves their WTP for HFMD vaccine.

Table 5: Parameter estimates for Seemingly unrelated bivariate probit model.

Explanatory variables	Single bounded function			Double bounded function		
	Coeff.	Robust Std error	p-value	Coeff.	Robust Std error	p-value
**Price**						
Start bid	-0.001	0.001	0.073*			
Follow-up bid				-0.003	0.001	0.000***
**Education**						
High School or below	0.035	0.285	0.903	-0.435	0.167	0.009***
Certificate or Diploma	0.725	0.258	0.005***			
Bachelor's Degree	0.648	0.237	0.006***			
**Household income**						
<RM1000				-0.716	0.282	0.011**
RM1001-RM3000	-0.144	0.143	0.314			
RM3001-RM5000						
RM5001-RM7000						
RM7001 & above	0.356	0.166	0.032**			
Constant	-0.004	0.318	0.988	0.212	0.146	0.146
RHO	0.717 (0.064)					

Wald test rho=0; Chi2(1) = 65.82; p-value>Chi2 = 0.000

***Statistically significant at 1%,
**statistically significant at 5% and
*statistically significant at 10%

The value of rho is estimated at 0.717, and it is positive signed and statistically is significantly different from zero in a Wald test, indicating that the outcome is related and some unobserved factors are positively related to both dependent variables. The confidence intervals around the median and mean WTP are estimates based on the Krinsky and Robb [17] procedure using 10,000 draws [18,19].

Table 6: Mean and Median of Krinsky and Robb procedure

	Mean=Median	95% lower bound	95% upper bound	ASL[a]
Single bounded	460.23	-453.52	1926.26	0.033
Double bounded	394.00	335.30	517.64	0.000

[a] ASL = Achieved Significance Level for testing H0: WTP<= 0 versus H1: WTP>0

The mean WTP estimated for single bounded was MYR460.23 (USD $102.17) and for double bounded was MYR394.00 (USD $87.47). The WTP for single bounded was higher compared to the double bounded response and indicates the efficiency of using the double-bounded approach.

Discussion

This study surveyed parents in Selangor, Malaysia to determine their WTP for HFMD vaccination. We found that the price of the vaccine, parents' education and income will influence their WTP for HFMD vaccination.

According to economic theory, the law of demand, as the price of goods increases the quantity demand of goods decreases. In this study, we found that as the HFMD vaccine bids increase, WTP of the parents' decreases. Currently, there is no study investigating WTP for HFMD, however, published studies for several types of vaccines also found the same outcome such as meningococcal conjugate vaccines [19]; dengue vaccine [21,22]; pneumococcal vaccine [23,24]; PCV-7 and influenza vaccine [25]; cholera vaccine [26]. This indirectly indicates that parents prefer the vaccine at the lowest price to vaccinate their children with HFMD vaccination.

This study found that those who had higher education tended to pay for at a low price more than those who had lower education. In single bounded responses, higher education had a positive and significantly affects the parents' WTP. This finding is in line with previous research [27]. However, parents with low education reduce their WTP as the bid increases in double bounded. This indirectly shows that low educated parents have a financial constraint to WTP for the HFMD vaccine. Moreover, the low income of parents also significantly influences parents WTP in this study. Furthermore, Olson et al. [28]. highlighted those financial constraints due to having a larger family decreased WTP for vaccination.

Household income significantly influences the parents WTP on HFMD vaccine in our findings. The same outcome seen in other studies, namely that household-month income influences WTP for vaccination [29,30]. Interestingly, parents who earn high-income increase WTP for HFMD vaccination at a given price (single bounded) and low-income parents reduce their WTP if price increases (double bounded). This indicates that parental income influences their decision on vaccination based on vaccine price. Lower WTP for vaccine among individuals with lower education and lower incomes indicates that when EV71 vaccination becomes available in

Malaysia, there could be substantial socioeconomic disparities in the absence of government subsidies.

The estimated mean value for HFMD vaccine in this study was RM394.00 for two doses, which is a higher value estimated for different vaccinations in Malaysia such as dengue vaccine MYR 39.21 per dose [31]; Hepatitis B vaccine RM303 for three doses [32] and coronavirus disease 2019 (COVID-19) vaccine RM134 for a single dose [33]; According to economic theory, the equilibrium of the price is determined by demand and supply of the product. This study suggests that the HFMD vaccine's price is the key determinant of parents' WTP for the HFMD vaccine if the vaccine is available in the future (demand side). Therefore, the manufacture (supply side) of the vaccine should use the cost-plus pricing setting [34], where the seller is paid a mark-up over the marginal cost and is able to produce the vaccine at the lowest cost. With that, HFMD vaccine would be available in the future at a more affordable price for a larger share of the population.

Conclusions

This study used a bivariate probit model to analyse the parents WTP on HFMD vaccination in Selangor, Malaysia. We found that 71.5% of the parents were willing to pay for HFMD vaccination. The estimated mean WTP for HFMD vaccine was RM394 (USD $87.47) for two doses. Moreover, the parents' education and monthly income are associated with WTP. The result shows that the higher the price (bid) of the vaccine, the lower the parents' WTP for HFMD vaccine. Moreover, parents' income and education significantly increase their WTP for HFMD vaccine. Based on our finding, there is a demand for the HFMD vaccine in Malaysia if available in the private market. However, the price of the vaccine should use a price discrimination strategy such as a sliding scale where a different price is charged based on the

parent's income. Moreover, awareness of HFMD should increase among the low educated parents to increase their WTP for HFMD if available in the private market in the future.

Acknowledgments

The authors would like to thank the questionnaire moderator committee from the Faculty of Health Sciences, Universiti Putra Malaysia: Dr. Rosliza Abdul Manaf, Department of Community Health, and Dr. Siti Zulaikha Binti Zakariah, Department of Medical Microbiology and Parasitology.

Fundings

This study was funded by Universiti Tunku Abdul Rahman Research Fund (UTARRF), grant number IPSR/RMC/UTARRF/2019-C1/Y03.

References

1. Chan YF, Sam IC, Wee KL, Abubakar S. Enterovirus 71 in Malaysia: a decade later. Neurology Asia. 2011;16(1):1-5.

2. Siegel K, Cook AR, La H. The impact of hand, foot and mouth disease control policies in Singapore: A qualitative analysis of public perceptions. Journal of public health policy. 2017 May;38(2):271-87.

3. Wang Y, Meng F, Li J, Li G, Hu J, Cao J, Yu Q, Liang Q, Zhu F. Willingness of parents to vaccinate their 6–60-month-old children with EV71 vaccines: a cross-sectional study in rural areas of northern Jiangsu Province. Human vaccines & immunotherapeutics. 2020 Jul 2;16(7):1579-85.

4. Chua KB, Kasri AR. Hand foot and mouth disease due to enterovirus 71 in Malaysia. Virologica Sinica. 2011 Aug;26(4):221-8.

5. Lun-hui XI, Xiao-jun LI, Kai JI, Jiang ZH, Peng GO, Wen-xia DO, Xing-tang YA. Investigation on enterovirus 71 vaccine immunization willingness of parents with

children aged 5 years in Baoshan district, Shanghai. 疾病监测. 2017 Dec 30;32(12):944-7.

6. Rajamoorthy Y, Tin OH, Taib NM, Munusamy S, Wagner AL, Mudatsir M, Khin AA, Anwar S, Sidique SF, Harapan H. Parents' knowledge and awareness towards hand foot mouth disease in Malaysia: A survey in Selangor. Clinical Epidemiology and Global Health. 2022 May 1;15:101027.
7. Rajamoorthy Y, Taib NM, Harapan H, Wagner AL, Aye KA, Munusamy S. Preventive Behaviors among Malaysian Parents towards Hand, Foot, and Mouth Diseases using Health Belief Model. IIUM Medical Journal Malaysia. 2022 Jan 1;21(1).

8. Krejcie RV, Morgan DW. Determining sample size for research activities. Educational and psychological measurement. 1970 Sep;30(3):607-10

9. Hanemann M, Loomis J, Kanninen B. Statistical efficiency of double-bounded dichotomous choice contingent valuation. American journal of agricultural economics. 1991 Nov 1;73(4):1255-63.

10. Cameron TA, Quiggin J. Estimation using contingent valuation data from a" dichotomous choice with follow-up" questionnaire. Journal of environmental economics and management. 1994 Nov 1;27(3):218-34.

11. Alberini A. Efficiency vs bias of willingness-to-pay estimates: bivariate and interval-data models. Journal of environmental economics and management. 1995 Sep 1;29(2):169-80.

12. Jeanty PW. Constructing krinsky and robb confidence intervals for mean and median willingness to pay (wtp) using stata. InSixth North American Stata Users' Group Meeting, Boston, August 2007 Aug 13 (pp. 13-14).

13. Entele BR. Analysis of households' willingness to pay for a renewable source of electricity service connection: evidence from a double-bounded dichotomous choice survey in rural Ethiopia. Heliyon. 2020 Feb 1;6(2):e03332.

14. Greene, W., 2000, Econometric Analysis, Prentice Hall.

15. Gu NY, Gai Y, Hay JW. The effect of patient satisfaction with pharmacist consultation on medication adherence: an instrumental variable approach. Pharmacy Practice (Internet). 2008 Dec;6(4):201-10.

16. Mitchell RC, Carson RT, Carson RT. Using surveys to value public goods: the contingent valuation method. Resources for the Future; 1989.

17. Krinsky I, Robb AL. On approximating the statistical properties of elasticities. The Review of Economics and Statistics. 1986 Nov 1:715-9.

18. Cooper JC. A comparison of approaches to calculating confidence intervals for benefit measures from dichotomous choice contingent valuation surveys. Land Economics. 1994 Feb 1:111-22.

19. Determann D, Korfage IJ, Lambooij MS, Bliemer M, Richardus JH, Steyerberg EW, de Bekker-Grob EW. Acceptance of vaccinations in pandemic outbreaks: a discrete choice experiment. PloS one. 2014 Jul 24;9(7):e102505.

20. Bishai D, Brice R, Girod I, Saleh A, Ehreth J. Conjoint analysis of French and German parents' willingness to pay for meningococcal vaccine. Pharmacoeconomics. 2007 Feb;25(2):143-54.

21. Palanca-Tan R. The demand for a dengue vaccine: a contingent valuation survey in Metro Manila. Vaccine. 2008 Feb 13;26(7):914-23.

22. Hadisoemarto PF, Castro MC. Public acceptance and willingness-to-pay for a future dengue vaccine: a community-based survey in Bandung, Indonesia. PLoS Negl Trop Dis. 2013 Sep 19;7(9):e2427.

23. Heinzen RR, Bridges JF. Comparison of four contingent valuation methods to estimate the economic value of a pneumococcal vaccine in Bangladesh. International journal of technology assessment in health care. 2008 Oct 1;24(4):481.

24. Sadique MZ, Devlin N, Edmunds WJ, Parkin D. The effect of perceived risks on the demand for vaccination: results from a discrete choice experiment. PloS one. 2013 Feb 8;8(2):e54149.

25. Hou Z, Chang J, Yue D, Fang H, Meng Q, Zhang Y. Determinants of willingness to pay for self-paid vaccines in China. Vaccine. 2014 Jul 31;32(35):4471-7.

26. Islam Z, Maskery B, Nyamete A, Horowitz MS, Yunus M, Whittington D. Private demand for cholera vaccines in rural Matlab, Bangladesh. Health Policy. 2008 Feb 1;85(2):184-95.

27. Tran BX, Than PT, Doan TT, Nguyen HL, Mai HT, Nguyen TH, Le HT, Latkin CA, Zhang MW, Ho RC. Knowledge, attitude, and practice on and willingness to pay for human papillomavirus vaccine: a cross-sectional study in Hanoi, Vietnam. Patient preference and adherence. 2018;12:945..

28. Olson D, Rick AM, Krager S, Lamb M, Asturias EJ. Vaccine demand and willingness-to-pay for arbovirus vaccines: a cross-sectional survey in rural Guatemala. The Pediatric infectious disease journal. 2018 Nov 1;37(11):1184-9.

29. Javan-Noughabi J, Kavosi Z, Faramarzi A, Khammarnia M. Identification determinant factors on willingness to pay for health services in Iran. Health economics review. 2017 Dec;7(1):1-6.

30. Sarmento TT, Godói IP, Reis EA, Godman B, Ruas CM. Consumer willingness to pay for a hypothetical chikungunya vaccine in Brazil and the implications. Expert review of pharmacoeconomics & outcomes research. 2019 Dec 14:1-8.

31. Yeo HY, Shafie AA. The acceptance and willingness to pay (WTP) for hypothetical dengue vaccine in Penang, Malaysia: a contingent valuation study. Cost Effectiveness and Resource Allocation. 2018 Dec;16(1):1-0.

32. Rajamoorthy Y, Radam A, Taib NM, Rahim KA, Munusamy S, Wagner AL, Mudatsir M, Bazrbachi A, Harapan H. Willingness to pay for hepatitis B vaccination in Selangor, Malaysia: A cross-sectional household survey. PLoS One. 2019 Apr 9;14(4):e0215125.

33. Wong LP, Alias H, Wong PF, Lee HY, AbuBakar S. The use of the health belief model to assess predictors of intent to receive the COVID-19 vaccine and willingness to pay. Human vaccines & immunotherapeutics. 2020 Sep 1;16(9):2204-14.

34. Lee BY, McGlone SM. Pricing of new vaccines. Human vaccines. 2010 Aug 1;6(8):619-26.

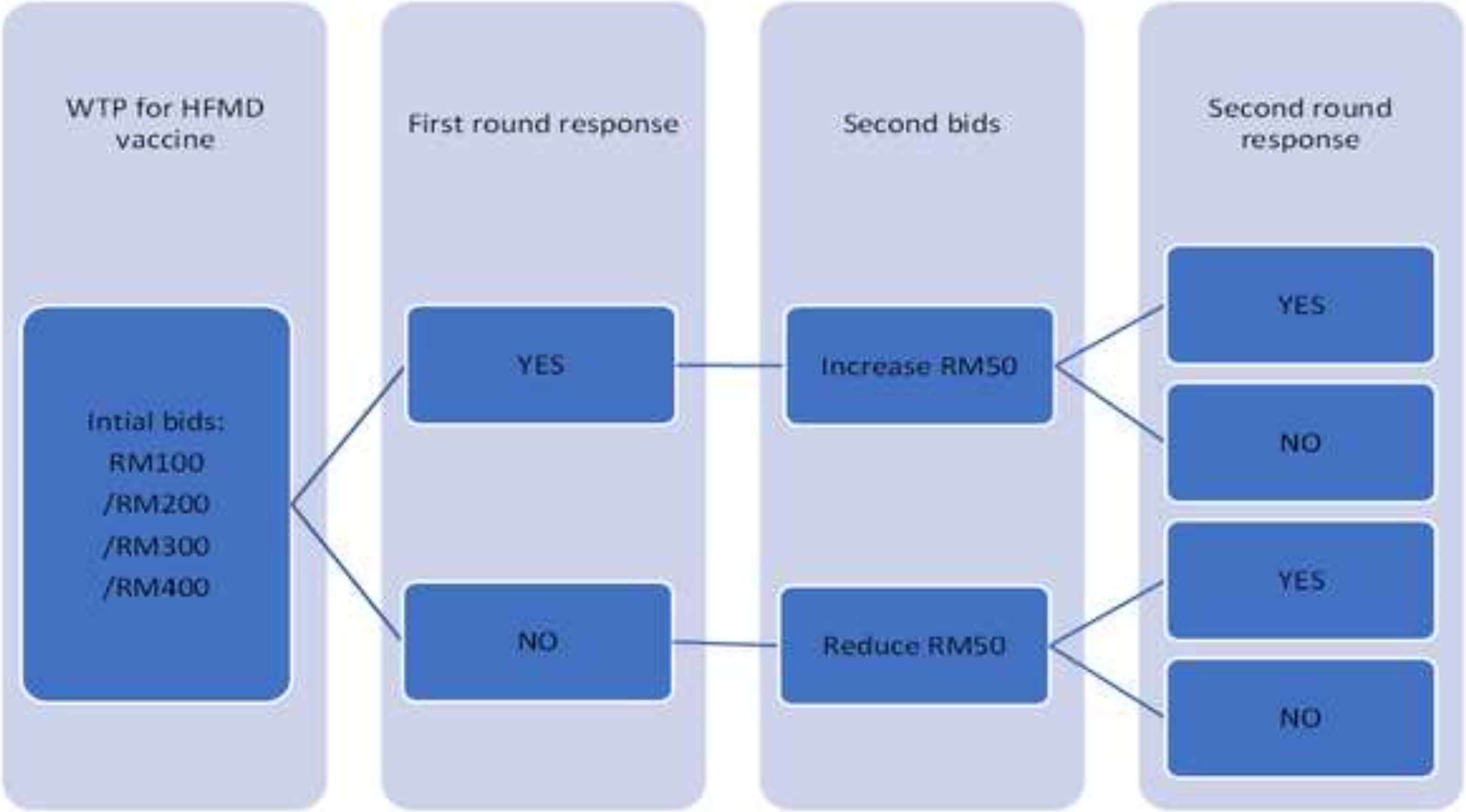

Fig 1: Flowchart of double bounded dichotomous choice

Data Review URL

Click here to download Data Review URL
http://figshare.com/s/3e6dd659b5efbc7a5e65
